# Intraperitoneal delivery of a novel drug‐like compound improves disease severity in severe and intermediate mouse models of Spinal Muscular Atrophy

**DOI:** 10.1038/s41598-018-38208-9

**Published:** 2019-02-07

**Authors:** E. Y. Osman, A. Rietz, R. A. Kline, J. J. Cherry, K. J. Hodgetts, C. L. Lorson, E. J. Androphy

**Affiliations:** 1 Department of Veterinary Pathobiology, Bond Life Sciences Center, College of Veterinary Medicine, University of Missouri, Columbia, MO 65201 USA; 2 Department of Dermatology, Indiana University School of Medicine, Indianapolis, IN 46202 USA; 3 Laboratory for Drug Discovery in Neurodegeneration, Brigham & Women’s Hospital, Harvard Medical School, Cambridge, MA 02139 USA

## Abstract

Spinal muscular atrophy (SMA) is an autosomal recessive neurodegenerative disorder that causes progressive muscle weakness and is the leading genetic cause of infant mortality worldwide. SMA is caused by the loss of *survival motor neuron 1* (*SMN1*). In humans, a nearly identical copy gene is present, called *SMN2*. Although *SMN2* maintains the same coding sequence, this gene cannot compensate for the loss of *SMN1* because of a single silent nucleotide difference in *SMN2* exon 7. *SMN2* primarily produces an alternatively spliced isoform lacking exon 7, which is critical for protein function. *SMN2* is an important disease modifier that makes for an excellent target for therapeutic intervention because all SMA patients retain *SMN2*. Therefore, compounds and small molecules that can increase *SMN2* exon 7 inclusion, transcription and SMN protein stability have great potential for SMA therapeutics. Previously, we performed a high throughput screen and established a class of compounds that increase SMN protein in various cellular contexts. In this study, a novel compound was identified that increased SMN protein levels *in vivo* and ameliorated the disease phenotype in severe and intermediate mouse models of SMA.

## Introduction

Spinal muscular atrophy (SMA) is the leading genetic cause of infant death. SMA is caused by genetic depletion or mutation of the telomeric *SMN1* gene. This results in the production of survival motor neuron (SMN) protein exclusively from the nearly identical, centromeric and inverted copy gene, called *SMN2*. Due to a critical C to T transition in exon 7, the majority of SMN transcripts and protein produced from *SMN2* are truncated due to an alternative splicing event that skips exon 7 with a small portion (~15%) being full-length SMN protein^1,2^. This C to T transition not only disrupts an exonic splicing enhancer site that usually allows splicing factor SF2/ASF to bind^3^, but it also generates a repressor element recognized by hnRNP A1^4^. Alternative splicing of exon 7 is a highly dynamic process involving an array of cis- and trans-acting factors, including ISSN1, a potent repressor of SMN exon 7 splicing. Recently, the U.S. FDA approved the first treatment for SMA. SPINRAZA™ (nusinersen) is a modified antisense oligonucleotide targeting the ISSN1 sequence^5–8^ that is approved for use in several countries. SPINRAZA™ is showing significant improvements in Type I SMA patients and earlier treatment results in better responses^9,10^. There is still a need for further therapeutic options, as the SMA population is clinically diverse and not all patients respond similarly to SPINRAZA™ treatment. Two small molecules in clinical trials also target SMN2 splicing, *Branaplam* (Novartis, NVS-1^11^, LM010) and *Risdiplam* (Roche & Genentech, RG-7916, PMID: 30044619). NVS analogs enhance SMN2 exon 7 splicing by stabilizing U1-pre-mRNA association proximal to the nGA motif at residues U5, C20, C21 and U22^11^. RG-7916 was not the original candidate; however, toxicity observed with RG-7800 in the eyes of monkeys led to the further clinical development of RG-7916^12,13^. RG-7916 is currently enrolled in two clinical trials and belongs to the SMN-C class of Roche/PTC splicing modulators. SMN-C5 was reported to cause chemical shift perturbations of 7 nucleotides in the 5′-ss of exon 7, and it binds to U1-pre-mRNA complex at Exonic Splice Enhancer 2 (ESE2) in exon 7, which is upstream of the NVS binding site^14^. SMN-C2 and SMN-C3 recently have been shown to bind to AGGAAG motif, which is almost identical to the ESE2, and leads to enhanced binding of FUBP1 and KHSRP^15^. Previously, we described the optimization of a small molecule series that increased SMN protein levels through a distinct mode-of-action. These compounds elevated SMN and increased SMN protein half-life^16–20^. We identified a novel isoxazole compound “4 m”^16,20^, which was active *in vitro* but demonstrated poor brain and plasma exposure following intraperitoneal (IP) administration to mice. During bioavailability structure-activity relationship (SAR) driven optimization of the isoxazole series, the amide linkage was shown to be associated with poor plasma stability, but was improved by changing the heterocycle and reversing the amide linkage^16^. The identified lead molecule compound (Compound 27), referred herein as “LDN-2014”, showed improved brain exposure after IP injection. This study represents a preliminary assessment of the effect of a novel compound, where we report the biological efficacy of LDN-2014 in two SMA animal models: the severe SMN∆7 and the intermediate *Smn*^*2B/−*^ mice.

## Results

### Analysis of SMN protein levels in the SMNΔ7 mouse model of SMA following LDN-2014 delivery

**Figure 1:**
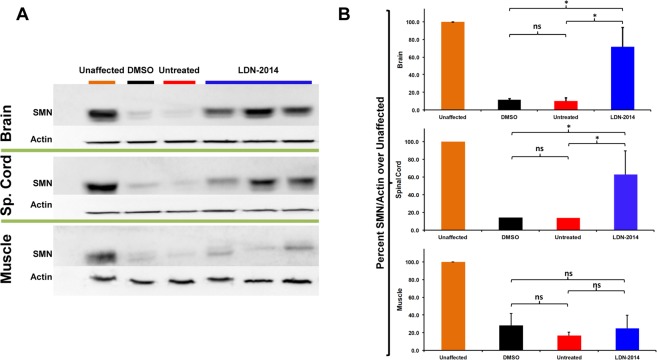
SMN expression after delivery of LDN-2014 in SMN∆7 mice. LDN-2014 (20 mg/kg, n = 3) was administered daily by IP injections. At P7 mice were sacrificed, tissues extracted and immunoblot for SMN and actin was performed. (**A**) Representative Immunoblot images of SMN protein levels in brain (top panel), spinal cord (middle panel), and muscle (bottom panel) of vehicle treated mice (DMSO, black) and compound (LDN-2014, blue)) vs. unaffected healthy mice (orange). (**B**) Quantification of SMN band signal intensity normalized over actin control, and expressed as a percentage from tissue from healthy animals. Data expressed as S.E.M.

To examine the activity of LDN-2014 *in vivo*, compound was dissolved in DMSO and delivered via IP injections into the well-established severe mouse model of SMA, “SMNΔ7” (*Smn*^−/−^; *SMN2*^+/+^; *SMNΔ7*^+/+^)^21^. DMSO was selected as the delivery vehicle due to limited aqueous solubility of this molecule (31 µM). LDN-2014 has an EC_50_ of 0.3 µM *in vitro*; results in a brain exposure of C_max_ = 41 μM and plasma levels of C_max_ = 23 μM with a plasma half-life (T_1/2_) of 2.2 hours following a single IP dose at 20 mg/kg (^16^, Supplementary Table 1A,B), rendering 20 mg/kg suitable for preliminary assessment of *in vivo* activity. Starting on P2, animals were injected daily with 20 mg/kg LDN-2014 and sacrificed on P7. Brain, spinal cord and skeletal muscle (gastrocnemius) were collected, solubilized and SMN protein expression measured by semi-quantitative immunoblot in comparison to the previously published transcriptional SMN inducer LDN-76^20^ (Fig. 1A,B). Administration of LDN-2014 significantly increased SMN protein expression in brain and spinal cord tissues compared to untreated and vehicle (DMSO) treated SMNΔ7 mice; however, there were no significant SMN increase in muscle (Fig. 1B).

### IP injections with LDN-2014 extend survival and improve the phenotype of severe SMA mice

**Figure 2:**
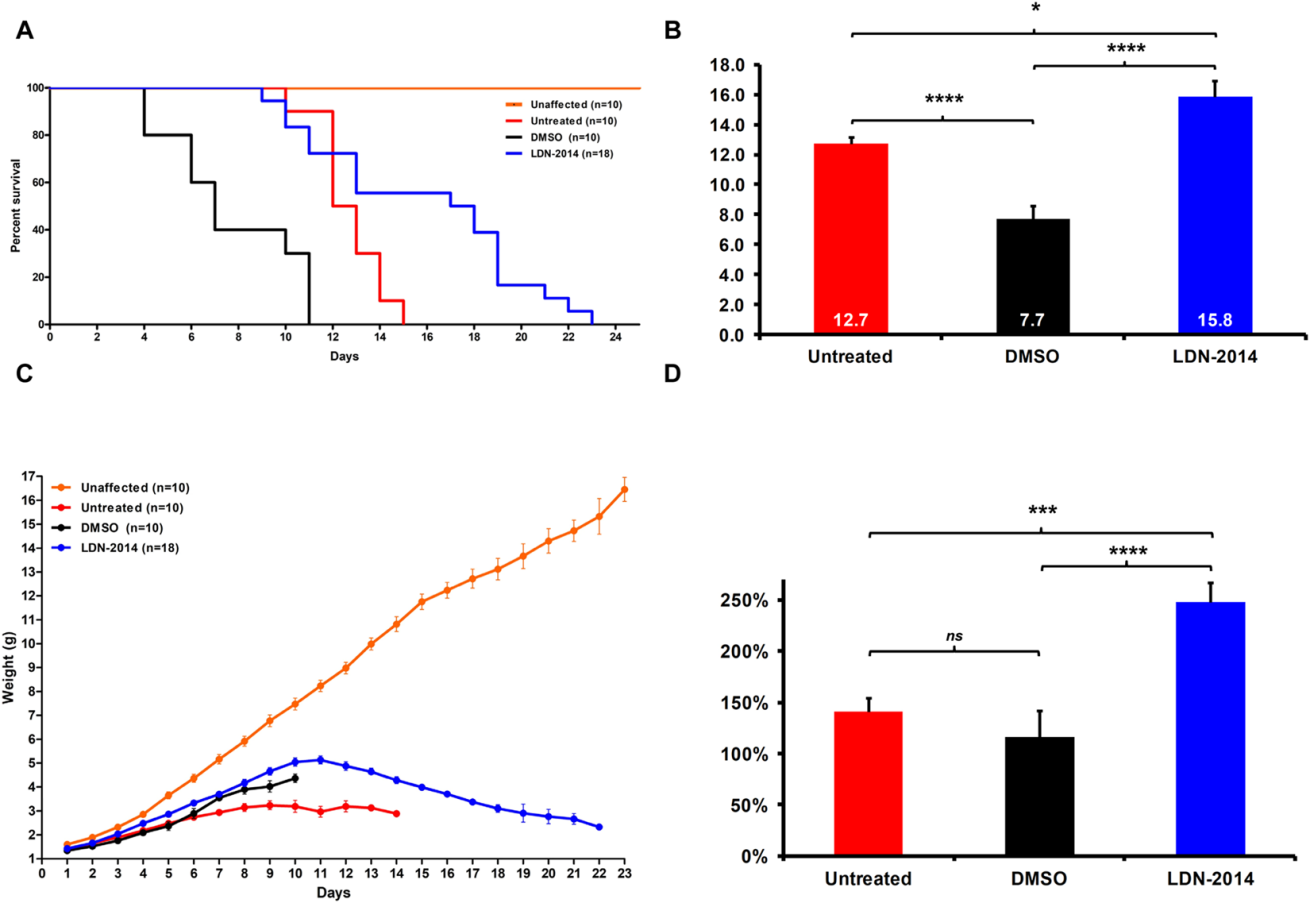
*In vivo* efficacy of LDN-2014 in SMN∆7 mice. LDN-2014 (20 mg/kg) or vehicle (DMSO) injections were administered by IP daily and bodyweight was recorded. (**A**) Kaplan-Meier survival curve of untreated SMA mice (n = 10) in comparison to DMSO (n = 10) and LDN-2014 (n = 19) treated mice. (**B**) Comparison of average survival time by Log-rank Mantel-Cox test. (**C**) Body weight curve in grams for DMSO (n = 10), LDN-2014 (n = 19), and untreated (n = 10) SMN∆7 mice. (**D**) Body weight expressed as percentage weight gain from “birth-to-peak”, from all cohorts. Statistical significance is represented by “*” where p ≤ 0.05; “**”p ≤ 0.01; “***”p ≤ 0.001; “****”p ≤ 0.001; ^“ns”^p > 0.05. Data expressed as S.E.M.

The increases in SMN protein led us to investigate whether LDN-2014 extended survival of SMNΔ7 mice. Treated animals received a single IP injection daily from P2 onward, corresponding to a daily dose of 20 mg/kg LDN-2014 and DMSO. The vehicle-only cohort (Fig. 2A,B) exhibited a median survival of 7.7 days compared to the 12.7 days for the untreated SMNΔ7 mice cohorts, an effect that we reported previously^20^. While the dose (2 ml/kg) of 100% DMSO is below the LD_50_ of 6.2 ml/kg^22^ observed in adult mice, this effect is enhanced in neonatal mice with progressive SMA phenotype. It is possible that the administration of DMSO exacerbates reported cardiovascular defects detected as early as P2 in SMNΔ7 mice^23,24^, as lower chronic doses of 100% DMSO also have been associated with toxicity in adult mice^25,26^. Administration of 100% DMSO resulted in cardiac failure, a pathology that has been observed in SMNΔ7 mice^23,27^. In contrast, LDN-2014 increased survival, with the longest lived animals living beyond 20 days (Fig. 2A,B). Compared to vehicle-treated, the LDN-2014-treated cohort lived significantly longer (~200%) and showed an increased lifespan of ~30% beyond untreated SMA mice. The increase in survival was accompanied by a significant weight gain in LDN-2014 treated mice compared to vehicle and untreated SMA animals (Fig. 2C), especially more evident past P9. To normalize for birth weight, body weight was represented as “birth-to-peak” weight, demonstrating that LDN-2014 treatment improves total body weight gain in SMA model mice (Fig. 2D).

**Figure 3:**
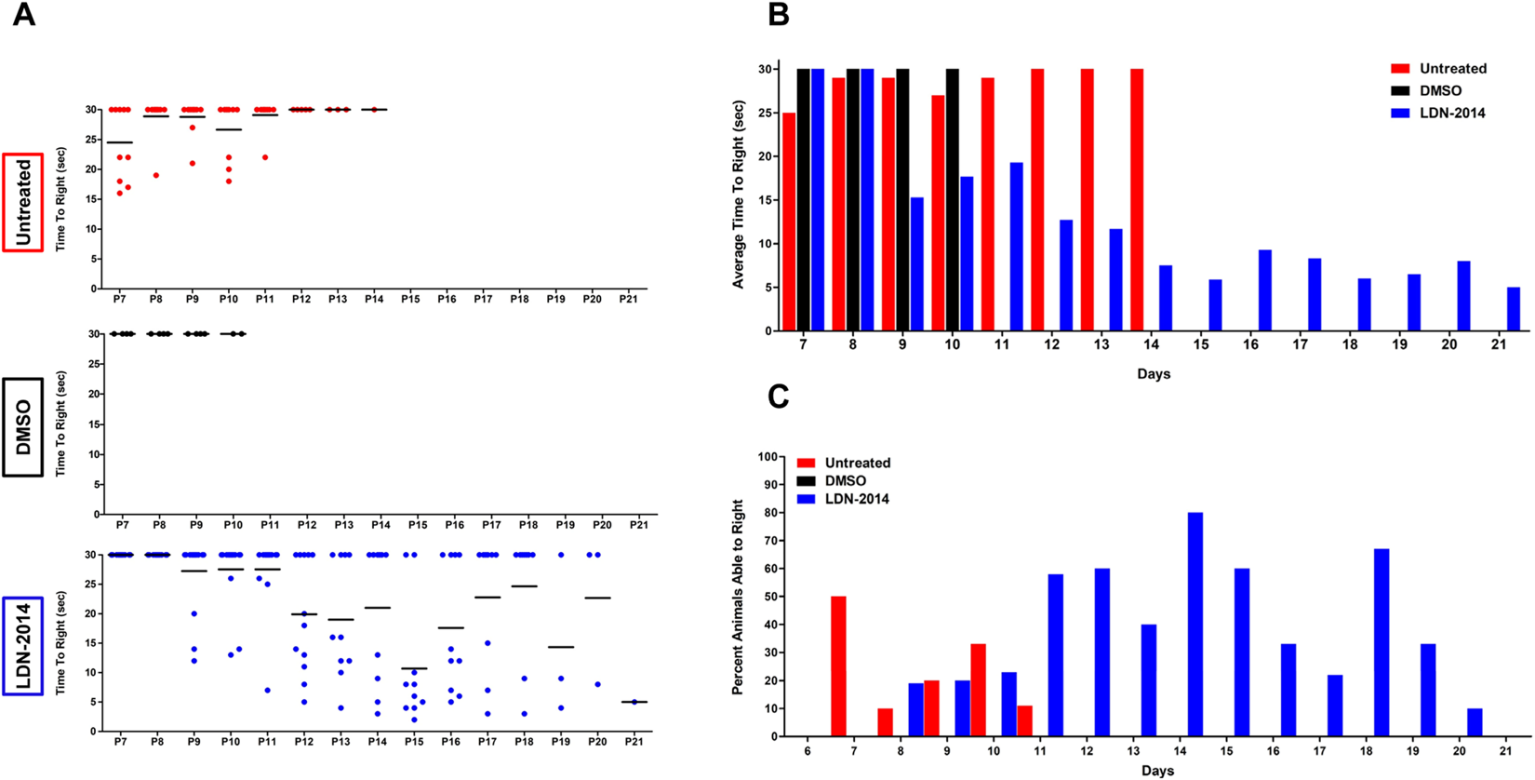
Gross motor function in drug-treated SMN∆7 mice. LDN-2014 (20 mg/kg) or vehicle (DMSO) injections were administered by IP daily. (**A**) Scatter plot representing the individual time-to-right of each treated animal (Untreated, DMSO, LDN-2014). (**B**) Bar graph showing the average time-to-right of each treatment group from ages of P7 through P21, where “*” represents a significant improvement compared to the control groups. (**C**) Cohorts expressed as a percentage of animals able to right themselves within 30 second. Data expressed as S.E.M.

To determine the impact of drug treatment upon gross motor function and strength, time-to-right assays were performed throughout the animals’ life span starting at P7 (Fig. 3). This time frame was selected as a starting point since this is the age unaffected mice are consistently able to right themselves from a prone position. Drug-treated SMA mice were able to right themselves starting at P9 and showed further improvement by P12, with additional improvements until P14. In contrast, vehicle-alone (DMSO) and untreated SMA mice rarely were able to right themselves within the 30 seconds limit. These improvements in motor function are consistent with the weight and survival improvements in LDN-2014 treated animals (Fig. 3).

### Delivery of LDN-2014 improves neuromuscular junction size and maturation in SMNΔ7 mice after treatment

**Figure 4:**
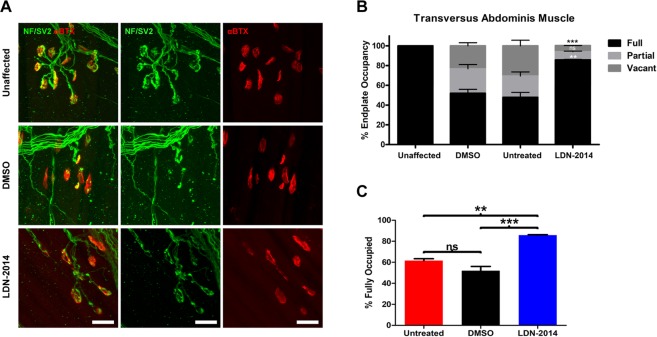
Improved neuromuscular junction pathology in treated SMN∆7 mice. LDN-2014 (n = 4, 20 mg/kg) or vehicle (n = 4) were administered by IP daily until P7. Unaffected control mice (n = 4) were sacrificed at same age. (**A**) Representative immunohistochemistry of NMJs of the individual cohorts. Neurofilament and synaptic vesicle are shown in green. Acetylcholine receptors are stained with α-Bungarotoxin (red). (**B**) Quantification of endplate occupancy of n = 4 per group. (**C**) Percentage of NMJs that are fully occupied in vehicle and LDN-2014 treated groups compared to the untreated cohorts. Data was analyzed using by one-way ANOVA and statistical significance is represented by “*”p ≤ 0.05; “**”p ≤ 0.01; “***”p ≤ 0.001; “****”p ≤ 0.001; ^“ns”^p > 0.05 and expressed as S.E.M.

To begin to examine the structural integrity of the motor units, neuromuscular junctions (NMJs) in the *transversus abdominis* (TVA) of vehicle and LDN-2014 treated SMN∆7 mice were analyzed by indirect immunofluorescence. The TVA was selected because it has previously been shown that neurons innervating this muscle are consistently vulnerable to disease and exhibit a significant degree of pathology in SMN∆7 mice and other mouse models of SMA. Pathological hallmarks include endplate morphology and denervation as quantified by a reduction in endplate occupancy^28–32^. Similarly to above, four mice per group were injected daily with LDN-2014 (20 mg/kg) or vehicle by IP delivery until P7. For control NMJ immunohistochemistry, unaffected mice (n = 4) were sacrificed at P7 for comparison. Pre-synaptic axons and nerve terminals were labeled with antibodies against total neurofilament and synaptic vesicle protein 2 (SV2) and post-synaptic acetylcholine receptors were labeled with rhodamine-conjugtaed α-Bungarotoxin (Fig. 4A). Administration of LDN-2014 significantly improved NMJ occupancy, with nearly 90% of endplates remaining fully occupied after drug-treatment compared to 50–60% in DMSO and untreated samples (Fig. 4B,C). Collectively, these results demonstrate that LDN-2014 increased SMN expression in disease-relevant tissues, and that this correlated with phenotypic improvements the motor units of SMA model mice, ultimately resulting in an extension in survival. Previous studies have shown that targeted SMN expression in motor neurons rescued the neuronal SMA phenotype in SMN∆7 mice^33^; however, this was not associated with a similar extension in survival. In contrast, an antisense oligonucleotide delivered by intracerebroventricular (ICV) injection significantly rescued the SMA phenotype in the SMN∆7 mice^34,35^. Therefore, we speculate that the increases in the periphery and the central nervous system result in the improvements in survival as well as gross motor function.

### Phenotypic improvements in intermediate SMA mice after LDN-2014 delivery

**Figure 5:**
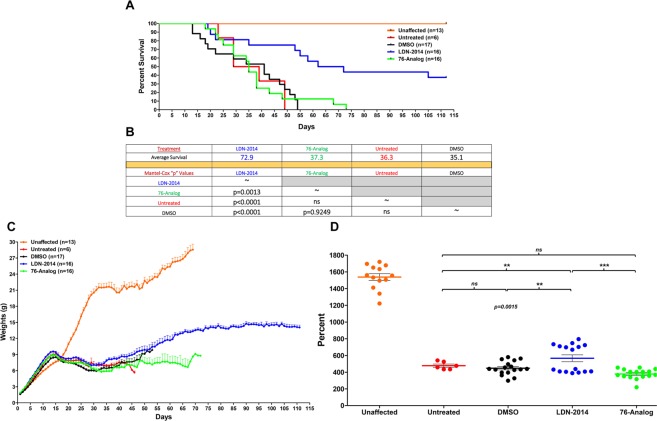
*In vivo* efficacy of LDN-2014 in an intermediate *Smn*^*2B/−*^ mouse model. LDN-2014 (5 mg/kg), 76-series analog (5 mg/kg) or vehicle (DMSO) injections were administered by IP q.a.d. (**A**) Kaplan-Meier survival curve of untreated SMA mice (n = 6) in comparison to vehicle (n = 17), LDN-2014 (n = 16), and 76-analog (n = 16) treated cohorts. (**B**) Summary table showing the average survival and Log-rank Mantel-Cox test “p” values for the various comparison. (**C**) Body weight measurements of untreated SMA mice (n = 6) in comparison to vehicle (n = 16), LDN-2014 (n = 16) and 76-analog (n = 16). (**D**) Body weight expressed as percentage weight gain from “birth-to-peak”, from all treated groups. Statistical significance is represented by “*” where p ≤ 0.05; “**”p ≤ 0.01; “***”p ≤ 0.001; “****”p ≤ 0.001; ^“ns”^p > 0.05. Data expressed as S.E.M.

Since SMA presents in a broad disease spectrum, we next wanted to determine whether LDN-2014 decreased disease severity in the *Smn*^*2B/−*^ intermediate model of disease. Using a similar delivery paradigm in which *Smn*^*2B/−*^ pups received LDN-2014 at 5 mg/kg from P2 via IP injections every other day. This dosing schedule was selected to limit the effects of long-term IP injections. Based upon the previous established PK profile after IP injection of LDN-2014, 5 mg/kg should result in a brain exposure of approximately C_max_ = 10 μM and a plasma exposure of C_max_ = 6 μM; both are well-above the *in vitro* EC_50_ of LDN-2014. In parallel, a cohort of *Smn*^*2B/−*^ mice received an LDN-76 analog, (5 mg/kg^36^,), as well as a cohort receiving vehicle (DMSO) every other day or mice that were left untreated. The vehicle and untreated *Smn*^*2B/−*^ animals had similar average life spans of approximately 36–37 days (Fig. 5A,B). Treatment with the LDN-76 analog resulted in a similar average lifespan compared to vehicle and untreated *Smn*^*2B/−*^ mice. In contrast, a significant extension in survival was observed in LDN-2014 treated *Smn*^*2B/−*^ animals, as the median survival for these animals was 73 days; however, more than 40% of the cohorts were alive when the experiment was halted at P110 (Fig. 5A,B). This was accompanied by improvements in weight gain, although these mice did not reach the maximum weights of unaffected control animals (Fig. 5C,D). The LDN-2014 treated mice maintained their weights throughout until P110, when the experiment was halted. The general appearance of LDN-2014 treated mice, normal posture and smooth hair coats, was improved compared to LDN-76 or untreated mice (Supplementary Fig. 1).

## Discussion

Clearly, increasing SMN protein is advantageous for SMA therapy, and exon 7 splicing and *SMN2* promoter activation are two well-examined modes-of-action. However, additional molecular pathways exist to elevate SMN levels; including approaches to prolong the half-life of SMN, as described for the proteasome inhibitor *Bortezomib*^37^, and the autophagy inhibitor 3-methyladenine^38^. Administration of *Bortezomib* to the severe SMN∆7 mice improved motor function and neuromuscular junction size, but did not extend survival. Due to low brain exposure, a follow-up study explored the combined treatment *Bortezomib* with a p-glycoprotein inhibitor to increase the CNS uptake. The combined treatment starting at P4 resulted in increased SMN protein levels in the brain and enhanced survival by 1.5 days in the severe SMN∆7 mice^39^. Administration of an autophagy inhibitor increased SMN protein levels and extended the lifespan by 1.5 days, while *Rapamycin*, an autophagy inducer, worsened the condition of the severe SMN∆7 mice^38^. Recently, administration of ML372, another novel small molecule that stabilizes the SMN protein through blockade of SMN ubiquitination^40^, resulted in increased SMN protein levels in brain, spinal cord and muscle and a survival extension of four more days. Although we do not know the mechanism by which LDN-2014 alters SMN protein levels, we previously found that LDN-2014 does not alter general proteasome or autophagy activity^17^. Current research to determine the pathway is ongoing, however, the mechanism(s) appears to be able to stabilize SMN protein derived from the human *SMN2* gene as well as the endogenous *Smn* (albeit mutated) gene. Examining the commonalities of these genes and/or the downstream regulators could provide insight into the mechanism as well as identifying a target for further drug development.

Based on these studies and our results, we propose that post-translational stabilization of the SMN protein may show a greater benefit in less severe SMA mice models, which could render them suitable to be of clinical benefit in combination with splicing modifiers and antisense oligonucleotides to prolong the time between treatments. In summary, we conclude that LDN-2014 is biologically active in two important animal models of SMA, and although limited by its solubility, it expands this mechanistically distinct avenue for the treatment of SMA.

## Methods

### Animal procedures and delivery of therapeutics

All animal experiments were carried out in accordance with protocols approved by the Animal Care and Use Committee of the University of Missouri. The animals had free access to water and food. Original breeder pairs of *Smn*^+/−^; *SMN2*^+/+^; *SMN∆7*^+/+^ mice were purchased from The Jackson Laboratory (JAX® Mice and Services, 610 Main Street Bar Harbor, ME 04609 USA). Litters were genotyped on the day of birth (P1) as previously described^41^, utilizing multiplex PCR on tail biopsy material with the following primer sets for the *Smn* gene: *mSmn*‐WT FWD (5′‐tctgtgttcgtgcgtggtgacttt‐3′) and *mSmn*‐WT REV (5′‐cccaccacctaagaaagcctcaat‐3′) and for the *Smn* knockout: *SMN1*‐KO FWD (5′‐ccaacttaatcgccttgcagcaca‐3′) and *SMN1*‐KO REV (5′‐agcgagtggcaacatggaaatcg‐3′). To control for litter size, treated and untreated control SMA mice were raised with two unaffected heterozygous siblings. The *Smn*^*2B/−*^ mice were bred from two colonies: *mSmn*^+/−^ heterozygotes (stock no. 006214; Jackson Laboratory) and *Smn*^*2B/2B*^ homozygotes. Intraperitoneal injections (IP) started the day after birth and continued daily or every other day as indicated. Drug was solubilized in 100% DMSO. Drug solutions and vehicle (DMSO) were injected in 10 µl per gram body weight. The Time‐to‐right (TTR) assessment started on P7 since healthy animals start to turnover at this time and the measurements were recorded daily. The test was performed on a flat surface and terminated after 30 seconds. A ‘Failure’ was recorded if an animal did not turn in this time frame. All TTR trials concluded on P20.

### Immunohistochemistry of NMJs

Animals from LDN-2014 treatment and vehicle groups were anesthetized at age P7 by inhalant Isoflurane, (1-chloro-2, 2,2-trifluoroethyl difluoromethyl ether; Fluriso^TM^, VetOne, Boise, ID 83705) followed by transcardiac perfusion with PBS solution and fixed with 4% paraformaldehyde (Sigma-Aldrich). *Transversus abdominis* muscle was rapidly dissected to yield whole-mount muscle preparations. Pre-synaptic axons and nerve terminals were stained with anti-neurofilament (1:2,000; catalog AB5539, Chemicon, EMD Millipore) and anti-SV2 (1:200 dilution, Chemicon®, MilliporeSigma, St Louis, MO, USA) for synaptic vesicles (NF/SV2). Acetylcholine receptors (AchR) were labeled with Alexa Fluor 594–conjugated α-Bungarotoxin (Life Technologies). Endplate occupancy was quantified to the following criteria: fully occupied is defined as complete coverage of the post-synaptic endplate by the branches of the pre-synaptic nerve terminal; partially occupied is defined as partial withdrawal of the pre-synaptic nerve terminal from the post-synaptic endplate; vacant is defined as complete withdrawal of the pre-synaptic nerve terminal from the post-synaptic endplate. Labeled pre-synaptic nerve terminals were visualized using 488 nm excitation and 520 nm emission optics, while endplates were visualized using 543 nm excitation and 590 nm emission optics. Quantification was performed blind, and incorporated a minimum of 50 NMJs from at least three distinct and randomly-selected fields of view per muscle per mouse (×40 objective; Leica DM5500 B, Leica Microsystems Inc.).

### Immunohistochemistry and quantification

SMN∆7 mice were treated with vehicle DMSO (vehicle) or LDN-2014 (20 mg/kg) for 7 days. Mice were sacrificed by brief exposure to general anesthetic (Isoflurane; Fluriso^TM^, VetOne, Boise, ID 83705) followed by cervical dislocation. Tissues (brain, spinal cord and gastrocnemius muscle) were extracted and immediately frozen in liquid nitrogen. The frozen tissues (~100 mg) were homogenized in JLB buffer (JLB: Jurkat lysis buffer; 50 mM Tris–HCl, pH 7.5, 150 mM NaCl, 20 mM NaH2(PO4), 25 mM NaF, 2 mM EDTA, 10% glycerol, 1% Triton X-100 and protease inhibitors (Roche, Indianapolis, IN, USA)). Equal amounts of protein were separated on 12% SDS–PAGE gels and transferred onto PVDF membranes. Membranes were blotted using anti-SMN monoclonal antibody (SMN specific monoclonal antibody (BD Biosciences, San Jose, CA, USA) diluted 1: 300 in TBST (Tris-buffered Saline Tween20 (10 mM Tris-HCl, pH 7.5, 150 mM NaCl, 0.2% Tween20)) in 1.5% dry milk. Blots were visualized by chemiluminescence on a Fujifilm imager LAS-3000 (FujiFilmUSA, Hanover Park, IL, USA) and the corresponding software. To verify equal loading the Westerns blots were then stripped using H_2_O_2_ for 30 minutes at room temperature and re-probed with anti-Actin antibody produced in rabbit (Cat. No. 5060; Sigma-Aldrich; Millipore-Sigma, St. Louis, MO 63103) and anti-rabbit horseradish peroxidase (HRP) secondary antibody (Cat. No. 711-035-152; Jackson ImmunoResearch Laboratories Inc.; West Grove, PA 19390), diluted 1:2000 and 1:10000, respectively. Western blots were performed in triplicates or more and representative blots are shown. The signal intensity was measured for each band on an immunoblot, normalized to the loading control, and the fold increase was determined in relation to the appropriate control. Data were quantified utilizing FUJI FILM MultiGauge v. 2.0 software.

### Statistical Methods

Survival was analyzed from the Kaplan-Meier survival curves using the log-rank Mantel-Cox test for survival comparisons. (Graph-Pad Prism v5.00; GraphPad Software, Inc., 7825 Fay Avenue, Suite 230, La Jolla, CA 92037 USA). A *p*-value of *p* < 0.05 was considered statistically significant. For weight gain and SMN expression (Western blots), statistical analysis were performed by GraphPad Prism as above (2-way ANOVA with Tukey’s multiple comparisons), by Student’s *t*-test using Excel 2013, version 15.0.4753.1003 and by IBM SPSS Statistics software. All data are expressed as standard error of mean (SEM) unless otherwise stated.

## Supplementary information

Supplemental Data
