# Supplementary material for: Intraperitoneal delivery of a novel drug‐like compound improves disease severity in severe and intermediate mouse models of Spinal Muscular Atrophy
Source: Sci Rep. 2019 Feb 7;9:1633. doi: 10.1038/s41598-018-38208-9 (PMC6367425; doi:10.1038/s41598-018-38208-9)
Supplement: Supplementary file 1 — Supplemental Data [file 41598_2018_38208_MOESM1_ESM.pdf]

Intraperitoneal delivery of a novel drug-like compound improves disease severity in severe and intermediate mouse models of Spinal Muscular Atrophy

Osman E.Y.[1, ‡], Rietz A.[2, ‡], Kline R. A.[1], Cherry J.J.[2], Hodgetts K.J.[3], Lorson C.L.[1] * and Androphy E.J.[2] *

[1] Department of Veterinary Pathobiology, Bond Life Sciences Center, College of Veterinary Medicine, University of Missouri, Columbia, MO 65201, USA

[2] Department of Dermatology, Indiana University School of Medicine, Indianapolis, IN 46202, USA

[3] Laboratory for Drug Discovery in Neurodegeneration, Brigham & Women's Hospital, Harvard Medical School, Cambridge, MA 02139, USA

* Indicates co-corresponding authorship

Supplementary Table 1A and B

Structure	Solubility (pH 7.4)	SMN2 EC50	Mouse µsome $T_{1/2}$	Human µsome $T_{1/2}$	Plasma stability
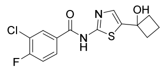	**31 µM**	**0.28 µM**	**39 min**	**>120 min**	**326 min**

Dose (mg/kg)	Plasma C_{max} (ng/mL)	Plasma $AUC_{0\text{-}24h}$ (ng.h/mL)	Plasma T_{max} (h)	Plasma $T_{1/2}$ (h)	Brain C_{max} (ng/mL)	Brain $AUC_{0\text{-}24h}$ (ng.h/mL)	Brain T_{max} (h)	Brain $T_{1/2}$ (h)	B/P
**20 mpk IP**	**7660**	**34291**	**0.5**	**2.2**	**13500**	**51310**	**0.5**	**1.9**	**1.5/1**

Supplementary Tables 1A and B LDN-2014 characteristics and pharmacokinetic properties

Tables are adapted from previously published data for compound LDN-2014 (compound **27,** [16]). (**A**) General characteristics of LDN-2014. (**B**) Mouse pharmacokinetic properties of LDN-2014

Supplementary Figure 1

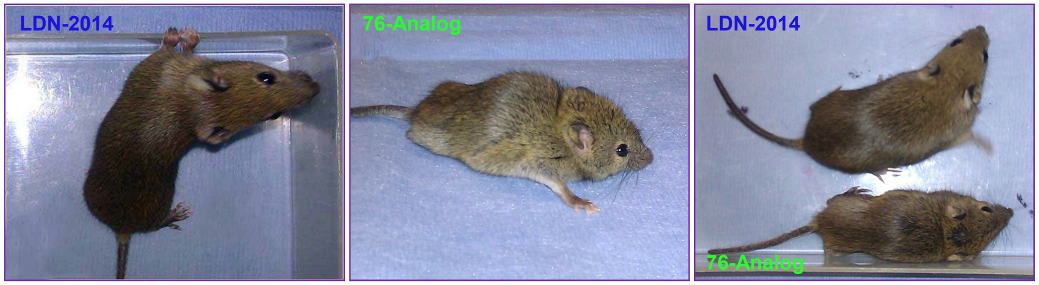

Supplementary Figure 1 *In vivo* efficacy of LDN-2014 in *Smn*$^{2B/-}$ mice

LDN-2014 (5 mg/kg), 76-series analog (5 mg/kg) or vehicle (DMSO) injections were administered by IP every other day. Representative images of general appearance of LDN-2014 treated mice in comparison to mice treated with the 76-series analog at age of P60. Development of spinal deformity (kyphosis) can be clearly seen in animals treated with the 76-series analog.
